# From spillover to preparedness: a One Health analysis of knowledge gaps in Nipah virus surveillance, prevention, and health system response

**DOI:** 10.3389/frhs.2026.1825624

**Published:** 2026-07-01

**Authors:** Sirwan Sleman, Omed I. Abid, Barham J. Abdullah, Basim A. Ali, Zaniar A. Abass, Masood B. Ameen

**Affiliations:** 1 College of Veterinary Medicine, University of Sulaimani, Sulaymaniyah, Iraq; 2 Masi Altuni Company, Sulaymaniyah, Iraq; 3 Sulaimani Private Veterinary Hospital, Sulaymaniyah, Iraq

**Keywords:** health system preparedness, Nipah virus, Nipah virus disease surveillance, Nipah virus ecology, Nipah virus One health, viral encephalitis, zoonotic spillover

## Abstract

**Background:**

Nipah virus (NiV) is a highly pathogenic zoonotic henipavirus that continues to pose a significant threat to global health security because of its high case fatality rate, ability to cause human-to-human transmission, and absence of licensed vaccines or specific therapeutics. Although more than two decades of research have advanced the understanding of NiV ecology, epidemiology, and clinical disease, major uncertainties remain regarding spillover mechanisms, environmental drivers, surveillance integration, and outbreak preparedness. These persistent knowledge gaps limit the ability to predict, prevent, and respond effectively to future outbreaks.

**Objective:**

This review synthesises current evidence on Nipah virus across the human–animal–environment interface and identifies critical One Health knowledge gaps that hinder surveillance, risk forecasting, and health system preparedness.

**Methods:**

A structured narrative review was conducted using literature published between 1998 and 2025. Evidence was identified through systematic searches of major scientific databases and relevant outbreak reports and was synthesised according to three interconnected One Health domains: animal reservoirs and viral ecology, environmental and anthropogenic drivers of spillover, and human infection, transmission, and health system response. Additional emphasis was placed on diagnostics, surveillance, governance, and socio-behavioural determinants.

**Results:**

A total of 210 studies were included in the final synthesis. The evidence base was strongly concentrated in bat ecology and human clinical research, whereas environmental modelling, social science, governance, and implementation research remained comparatively limited. Key gaps included incomplete understanding of viral shedding dynamics in reservoir hosts, limited integration of climate and land-use data into spillover prediction models, insufficient evidence on asymptomatic infection and transmission patterns, restricted access to rapid diagnostics, fragmented surveillance systems, and weak cross-sectoral coordination. Significant deficiencies were also identified in governance frameworks, ethical preparedness, and evaluation of community-based prevention strategies.

**Conclusion:**

Current Nipah virus preparedness remains constrained by fragmented knowledge and limited operationalisation of the One Health approach. Strengthening integrated surveillance systems, ecological forecasting, decentralised diagnostics, implementation research, and cross-sector governance will be essential for improving outbreak prevention and response. Addressing these priorities can enhance resilience against Nipah virus and provide a framework for preparedness against future emerging zoonotic threats.

## Graphical Abstract

**Figure:**
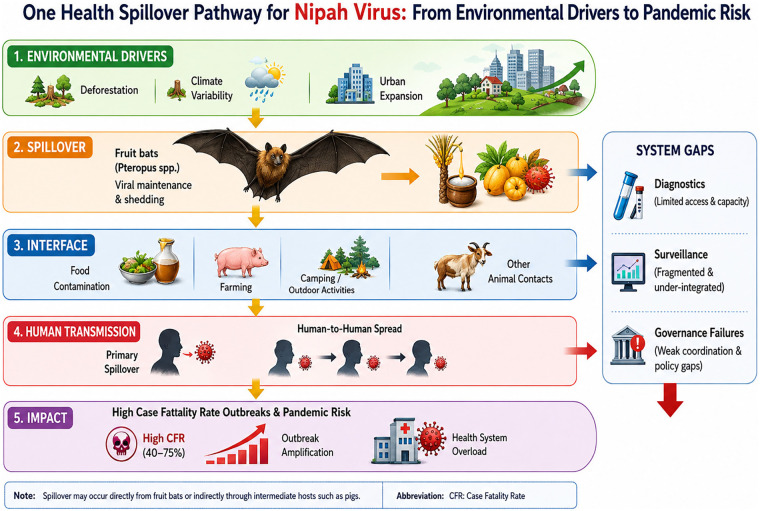
This visual summary illustrates the climate-driven One Health pathway of Nipah virus emergence, beginning with environmental disruption and bat viral maintenance, progressing through human exposure and transmission, and culminating in high case fatality outbreaks. It highlights critical system gaps—including diagnostics, surveillance, and governance—that amplify spillover risk and hinder effective prevention.

## 1 Introduction

Zoonotic viruses continue to pose a significant threat to global health security, particularly where human activities intersect with wildlife and environmental systems. Nipah virus (NiV), a member of the genus *Henipavirus*, represents a paradigmatic example of a zoonotic pathogen emerging from complex interactions between humans, animals, and ecosystems (1, 2). Since the first recognised outbreak in Malaysia (1998–1999), NiV has caused recurrent outbreaks in Bangladesh and India, with case fatality rates ranging from 40% to over 75% (3–7). In addition, NiV-like outbreaks have been reported outside these core regions, including the Philippines (2014), where infection associated with horse exposure suggested a broader ecological and geographic risk (8). These findings underscore the potential for wider regional emergence beyond South Asia.

Nipah virus is an enveloped, pleomorphic, negative-sense single-stranded RNA virus belonging to the family *Paramyxoviridae* and genus *Henipavirus* (9, 10). The viral genome is approximately 18.2 kb in length and encodes six major structural proteins, namely, the nucleocapsid (N), phosphoprotein (P), matrix (M), fusion (F), attachment glycoprotein (G), and large polymerase (L) proteins (9). The attachment (G) and fusion (F) glycoproteins facilitate viral entry through binding to ephrin-B2 and ephrin-B3 receptors, which are widely expressed in endothelial, neuronal, and respiratory tissues, thereby contributing to the broad host range and high pathogenicity of the virus (9, 10).

Following exposure, Nipah virus initially replicates in respiratory and epithelial tissues before disseminating systemically through the bloodstream. Endothelial cells and neurons are primary targets, resulting in widespread vasculitis, respiratory disease, and severe encephalitis (9, 11). Transmission occurs through multiple pathways, including direct spillover from infected fruit bats (*Pteropus* spp.), consumption of bat-contaminated food products such as raw date palm sap, contact with infected intermediate hosts—particularly pigs—and human-to-human transmission via respiratory secretions and close physical contact (2, 12, 13). Human-to-human transmission has been repeatedly documented during outbreaks in Bangladesh and India, particularly in healthcare and household settings (12). Although the estimated basic reproduction number (R_0_) is generally below 1, localised transmission clusters and nosocomial amplification events demonstrate the potential for sustained transmission under favourable conditions (6, 12).

Unlike some zoonotic viruses that show limited human-to-human transmission, NiV has demonstrated human-to-human transmission, especially among hospitalised patients and households (12). Recent epidemiological investigations have highlighted the continued risk of nosocomial amplification and the importance of rapid infection prevention measures during outbreaks (6, 12). Clinical manifestations range from acute febrile illness and respiratory disease to severe encephalitis and delayed neurological relapse, underscoring both its pathogenic versatility and the challenges of clinical recognition (9, 11, 14). Despite the danger posed by the zoonotic virus, no vaccines or drugs are available, and control of outbreaks primarily relies on non-pharmacological interventions (10).

The epidemiology of Nipah virus is intrinsically linked to fruit bat reservoirs, primarily *Pteropus* species, which maintain the virus asymptomatically across wide geographic ranges (15). Recent ecological modelling studies suggest that climate change and land-use shifts may alter bat distribution and future spillover zones, potentially expanding geographic risk (16, 17). However, spillover events remain sporadic and poorly predictable, indicating that ecological disruption, climate variability, agricultural intensification, and socio-cultural behaviours act as interacting drivers rather than isolated determinants. Activities such as deforestation, expansion of livestock farming, and consumption of raw date palm sap have repeatedly been implicated, yet these factors are often examined independently rather than within frameworks of integrated systems (2, 13).

One Health is a concept that considers the inter-relationship between human, animal, and environmental health. It is a tool through which the emergence of Nipah virus infections can be addressed. However, the application of the One Health concept for the Nipah virus is still lacking. For example, the surveillance systems and data sharing on the virus are not integrated. In addition, the social and behavioural sciences are not incorporated into studies (18). Most studies on the Nipah virus have been conducted with a narrow focus on virology, clinical outcomes, and ecology, without combining the results from different fields to highlight the gaps.

Accurate and timely diagnosis is fundamental for surveillance, outbreak detection, and containment of emerging zoonotic diseases such as Nipah virus infection. Current laboratory confirmation relies primarily on genotypic approaches, including real-time reverse-transcription polymerase chain reaction (RT-PCR), conventional RT-PCR, genome sequencing, and metagenomic analysis, which enable sensitive detection and molecular characterisation of viral strains. Phenotypic methods, including virus isolation, immunohistochemistry, and serological assays such as enzyme-linked immunosorbent assays (ELISA), provide complementary information regarding viral circulation and host immune responses (10). Despite advances in diagnostic technologies, access to rapid and decentralised testing remains limited in many endemic and resource-constrained settings. Furthermore, substantial gaps persist in integrating ecological, environmental, epidemiological, and health system evidence into a coherent One Health framework capable of supporting predictive surveillance and preparedness.

**Figure 1:**
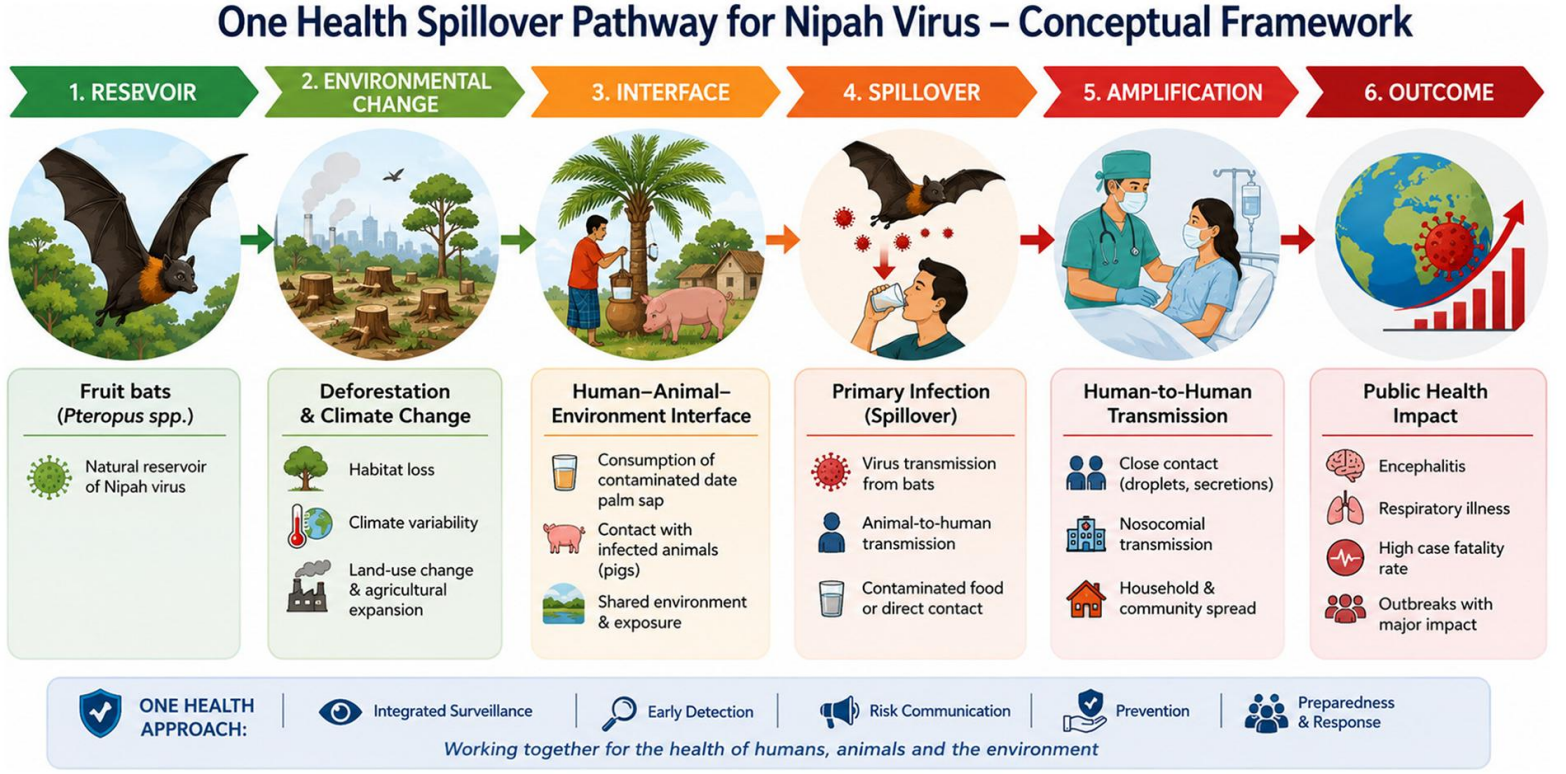
One Health Spillover pathway for Nipah virus (conceptual framework). This figure illustrates the sequential progression of Nipah virus emergence from bat reservoirs through environmental disruption and human–animal interfaces to spillover and amplification in healthcare and household settings. It highlights how ecological stressors and human exposure pathways converge to produce high-fatality outbreaks.

This review aims to identify knowledge gaps related to the Nipah virus within the One Health framework. Specifically, the review synthesises evidence across animal reservoirs, environmental drivers, transmission pathways, diagnostics, surveillance systems, and health system preparedness to identify critical areas where knowledge remains fragmented, insufficient, or poorly translated into practice. By synthesising evidence from ecological, environmental, and human health domains, it focuses on areas where current understanding is lacking or misaligned. The goal is to inform future research, guide policy development, and improve outbreak preparedness for this significant emerging zoonosis. The conceptual One Health spillover pathway underpinning this review is illustrated in Figure 1.

## 2 Methods

### 2.1 Study design and conceptual framework

This review was conducted in alignment with the Preferred Reporting Items for Systematic Reviews and Meta-Analyses (PRISMA 2020) guidelines. Although a narrative synthesis approach was adopted because of the heterogeneity of study designs, a structured screening process was implemented.

Rather than aiming for exhaustive quantitative pooling, the review prioritised conceptual integration and gap identification, which is appropriate for complex zoonotic systems characterised by heterogeneity in data sources and study designs.

The One Health framework was operationalised through three interlinked domains:

1. Animal reservoirs and viral ecology,
2. Environmental and anthropogenic drivers, and
3. Human transmission, clinical burden, and health systems response.

Cross-cutting dimensions included socio-behavioural factors, surveillance capacity, diagnostics, and governance.

### 2.2 Literature search strategy

The screening process followed a structured multi-stage approach. Titles and abstracts were independently reviewed for relevance, followed by full-text assessment against predefined inclusion criteria. Discrepancies were resolved through consensus. The review process included identification, duplicate removal, title and abstract screening, full-text eligibility assessment, and final inclusion according to predefined criteria.

A systematic literature search was conducted across PubMed/MEDLINE, Scopus, Web of Science, Embase, and Google Scholar. Additional records were identified through manual screening of reference lists from relevant reviews, outbreak reports, and international agency documents, including publications from the World Health Organization (WHO). Searches covered the period from January 1998 (corresponding to the first recognised Nipah virus outbreak) through December 2025.

Search terms combined controlled vocabulary and free-text keywords related to Nipah virus ecology, transmission, surveillance, and One Health. Representative search terms included the following: “Nipah virus”, “Henipavirus”, “Pteropus”, “fruit bat”, “spillover”, “zoonotic transmission”, “human-to-human transmission”, “One Health”, “surveillance”, “outbreak preparedness”, “diagnostics”, “encephalitis”, “deforestation”, “climate change”, and “date palm sap”. Boolean operators (AND, OR) and truncation strategies were applied to maximise search sensitivity. A representative search string was (“Nipah virus” OR Henipavirus) AND (spillover OR transmission OR surveillance OR preparedness OR ecology OR “One Health”).

**Figure 2:**
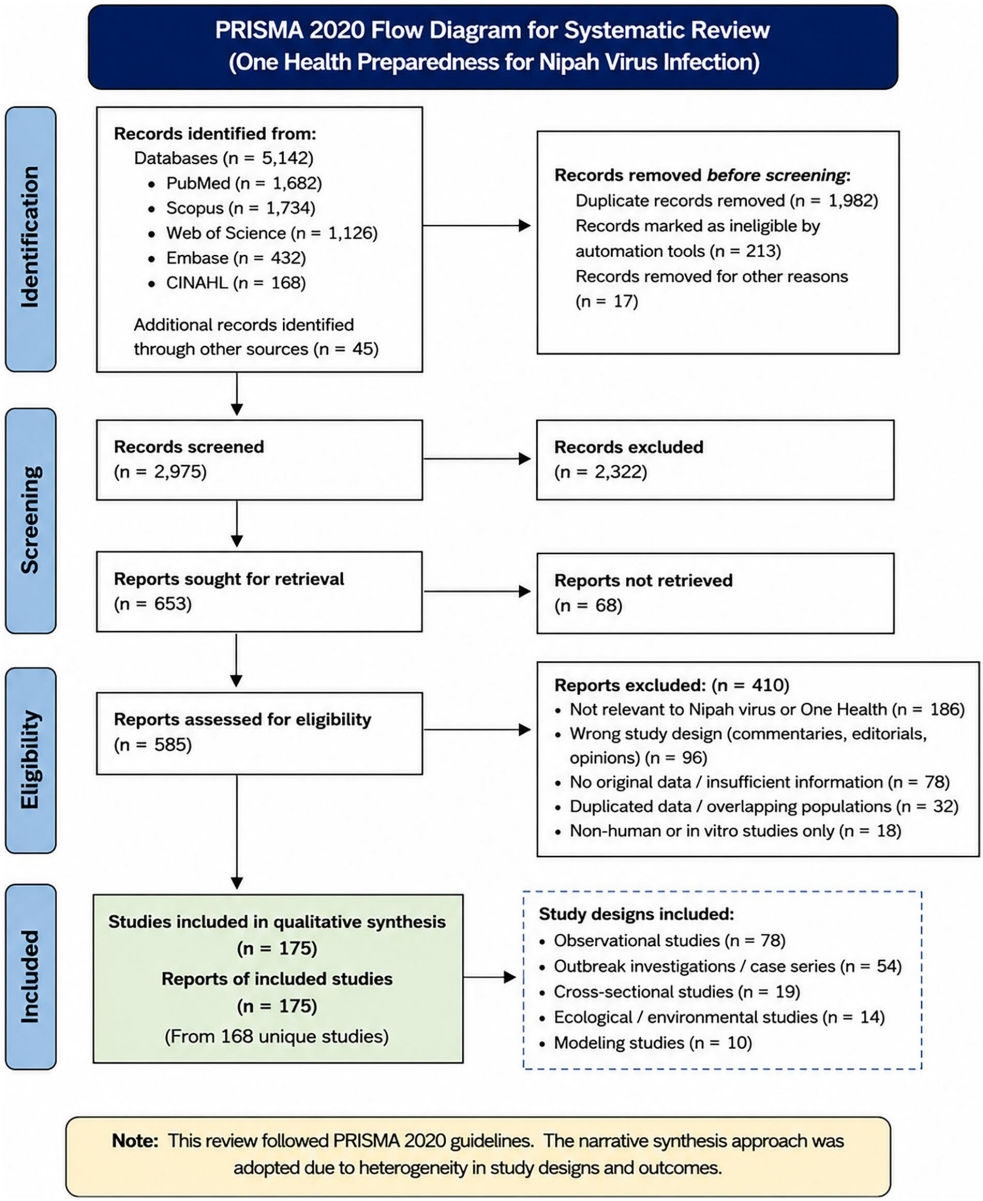
The PRISMA 2020 flow diagram. It summarises study identification, screening, eligibility, and inclusion.

A total of 5,721 records were identified through database searching and other sources. After removal of duplicates and ineligible records (*n* = 2,199), 3,522 records were screened, of which 857 reports were sought for retrieval and 743 full-text articles were assessed for eligibility. Following full-text review, 210 studies were included in the final synthesis (Figure 2).

Given the heterogeneity of included study designs (ecological, observational, and outbreak investigations), a formal quantitative risk-of-bias assessment was not feasible. However, study limitations, methodological variability, and overall evidence strength were systematically evaluated and incorporated into the synthesis.

### 2.3 Eligibility criteria

Studies were included if they

1. Investigated Nipah virus infection in humans, animals, or both;
2. Examined ecological, environmental, climatic, agricultural, or socio-behavioural determinants of spillover;
3. Reported surveillance, diagnostic, outbreak investigation, preparedness, or response data;
4. Addressed One Health, zoonotic transmission, reservoir ecology, or health system preparedness; and
5. Were published in peer-reviewed journals, official outbreak reports, or authoritative international agency documents.

Studies were excluded if they

1. Were non-English publications without accessible translations;
2. Focused exclusively on molecular or laboratory characterisation without ecological, epidemiological, surveillance, or public health relevance;
3. Consisted solely of editorials, commentaries, opinion pieces, conference abstracts, or letters without original data or systematic synthesis;
4. Reported insufficient methodological information for interpretation; or
5. Duplicated data already included in more comprehensive reports.

### 2.4 Data extraction and synthesis

Data were extracted into a standardised matrix capturing study location, domain (human, animal, environment), methodology, principal findings, and limitations. Thematic synthesis was then conducted iteratively, with particular attention to areas of inconsistency, absence of evidence, or failure of translation into policy or practice. Identified gaps were mapped across domains to reveal structural weaknesses in the current One Health implementation.

### 2.5 Operationalising One Health in Nipah virus preparedness

Translating the One Health framework into practice requires functional integration across surveillance, data systems, and response mechanisms. Several operational models can be identified:

1. **Integrated surveillance systems:** Linking animal (bat and livestock), environmental (land-use, climate), and human health data into unified early warning platforms.
2. **Cross-sectoral data sharing platforms:** Real-time data exchange between ministries of health, agriculture, and environment to enable coordinated responses.
3. **Decentralised diagnostic networks:** Expansion of regional laboratory capacity with rapid diagnostic tools to reduce delays in outbreak confirmation.
4. **Joint outbreak response units:** Multidisciplinary teams combining epidemiologists, veterinarians, ecologists, and social scientists.
5. **Community-based risk reduction:** Behavioural interventions (e.g., safe date palm sap consumption) integrated with surveillance systems.

These models demonstrate that effective One Health implementation requires institutional coordination, shared infrastructure, and sustained investment, rather than conceptual alignment alone.

## 3 Results

Across the 210 studies included in the final synthesis, evidence was unevenly distributed across One Health domains. Animal reservoir ecology and human clinical investigations accounted for the largest proportion of available evidence, whereas environmental modelling, governance, surveillance integration, and implementation research were comparatively under-represented. Three overarching findings emerged: (i) significant progress has been made in understanding reservoir ecology and transmission pathways; (ii) major uncertainties remain regarding spillover prediction, viral shedding dynamics, and asymptomatic infection; and (iii) surveillance systems, diagnostic preparedness, and cross-sectoral coordination remain insufficiently integrated to support proactive outbreak prevention.

### 3.1 Animal reservoirs and viral ecology

Fruit bats of the genus *Pteropus* are well established as natural reservoirs of Nipah virus, with widespread serological and molecular evidence across South and Southeast Asia (10). However, recent longitudinal and experimental studies have significantly advanced the understanding of viral dynamics, particularly regarding shedding and immune responses.

For example, Plowright et al. demonstrated that seasonal pulses of viral shedding are linked to nutritional stress and reproductive cycles, influencing spillover risk (19). Similarly, field studies have shown episodic shedding events rather than continuous viral excretion, complicating prediction models (20).

Despite these advances, key uncertainties remain, including the duration of immunity, interspecies transmission potential, and strain-specific virulence differences. Evidence from pig amplification during the Malaysian outbreak confirms livestock as critical intermediate hosts, yet routine surveillance in livestock populations remains limited, restricting early warning capacity (13).

Overall, the strongest evidence exists for bat reservoir identification and transmission ecology, whereas viral shedding dynamics, immunity, and environmental triggers remain among the most important unresolved questions.

### 3.2 Environmental and anthropogenic drivers

Environmental and anthropogenic drivers—including deforestation, agricultural intensification, and climate variability—have been repeatedly associated with Nipah virus spillover (15, 17). Empirical and modelling studies indicate that land-use change alters bat foraging behaviour and increases human–wildlife interface contact, thereby elevating spillover risk (7, 16).

Climate variability has also been linked to changes in fruiting phenology and bat nutritional stress, which, in turn, influence viral shedding dynamics (15, 19). However, most studies examine these drivers in isolation, and integrated eco-epidemiological models remain scarce, limiting predictive capacity.

A major gap lies in the lack of harmonised datasets linking land-use change, bat movement ecology, and outbreak timing. Consequently, predictive spillover modelling remains limited, undermining early warning systems.

Collectively, the findings indicate that environmental disruption is consistently associated with spillover risk; however, the lack of integrated eco-climate models substantially limits predictive capacity. Key environmental and anthropogenic drivers implicated in spillover are summarised in Table 1.

**Table 1:** Environmental and anthropogenic drivers of Nipah virus spillover.

Driver	Mechanism	Evidence type	Research limitation
Deforestation	Alters bat foraging	Observational	No causal quantification
Agricultural expansion	Human–bat contact	Case studies	Context-specific
Climate variability	Stress-induced shedding	Ecological inference	Sparse climate-linked data
Urbanisation	Habitat overlap	Remote sensing	Limited temporal linkage
Food practices	Sap contamination	Epidemiological	Intervention durability unknown

### 3.3 Human infection and transmission dynamics

Human-to-human transmission of the Nipah virus has been well documented, particularly in healthcare and household settings. Evidence from Bangladesh outbreaks demonstrates significant nosocomial amplification, with transmission occurring through close contact with respiratory secretions (2, 12).

Importantly, Sazzad et al. reported nosocomial transmission among healthcare workers, highlighting inadequate infection control measures as a key risk factor (21).

More recent outbreak investigations, including reports from India (2023–2026), further confirm the continued vulnerability of healthcare settings, particularly in resource-limited environments where infection prevention infrastructure and rapid diagnostics are insufficient. However, transmission efficiency, duration of infectivity, and the role of asymptomatic infections remain incompletely understood, representing critical gaps for outbreak control strategies.

The available evidence confirms the importance of human-to-human transmission in outbreak amplification, but significant uncertainties remain regarding asymptomatic infections, transmission heterogeneity, and outbreak forecasting.

### 3.4 Surveillance, diagnostics, and health systems preparedness

Health system preparedness for Nipah virus outbreaks remains uneven and underdeveloped, particularly in endemic regions.

**Workforce capacity:**

There is a shortage of trained personnel in outbreak detection, infection prevention and control (IPC), and zoonotic disease management. Limited cross-sector training further weakens One Health implementation.

**Laboratory infrastructure:**

Diagnostic capacity is highly centralised, often restricted to biosafety level-3/4 laboratories, resulting in delays in confirmation and response. Decentralised and rapid diagnostic tools remain limited.

**Infection prevention and control (IPC):**

Nosocomial transmission continues to occur because of inadequate IPC measures, including insufficient personal protective equipment, training gaps, and delayed case recognition.

**Surveillance systems:**

Current systems are largely reactive, with minimal integration of animal and environmental surveillance data. This fragmentation reduces early warning capability.

**Governance and coordination:**

Weak intersectoral coordination, unclear roles, and limited data-sharing frameworks hinder effective outbreak response (6, 10).

**Regional variability:**

Preparedness capacity varies significantly between countries and regions, influenced by resource availability, governance structures, and prior outbreak experience.

Overall, preparedness remains largely reactive rather than preventive, with fragmented surveillance systems and unequal access to diagnostic and response capacities representing major barriers to operational One Health implementation.

Tables 2, 3 not only summarises knowledge gaps but also prioritises them based on potential impact on outbreak prevention and feasibility of intervention, enabling clearer guidance for policy and research.

**Table 2:** One Health evidence strength and critical knowledge gaps in Nipah virus research.

One Health Domain	Evidence strength	Key evidence-based findings	Critical knowledge gaps
Animal reservoirs (15, 19, 20)	Moderate–high	*Pteropus* bats are confirmed natural reservoirs	Viral shedding duration, immunity, environmental triggers
Livestock interface (13)	Low–moderate	Pigs can function as amplification hosts	Limited livestock surveillance and early warning systems
Environmental drivers (1^4^–^17^)	Low	Deforestation, land-use change, and climate variability are associated with spillover risk	Lack of integrated eco-climate predictive models
Human transmission (2, 12, 21)	Moderate	Human-to-human transmission is well documented	Superspreading events and asymptomatic transmission remain poorly characterised
Clinical spectrum (9, 11, 14)	Low–moderate	Severe encephalitis dominates reported cases	Limited data on mild, asymptomatic, and long-term outcomes
Diagnostics (10)	Low	RT-PCR remains the primary diagnostic standard	Limited availability of field-deployable diagnostic platforms
Social and behavioural factors (2, 13)	Low	Foodborne exposure and caregiving practices contribute to risk	Limited evaluation of intervention effectiveness
Governance and ethics (18)	Very low	One Health frameworks are increasingly recognised	Lack of standardised governance and ethical preparedness frameworks

**Table 3:** One Health knowledge gaps in Nipah virus research.

Domain	Evidence strength	Key findings	Critical knowledge gaps	Implications
Animal reservoirs	Moderate	*Pteropus* bats are the primary reservoirs	Viral shedding duration, immunity, and stress effects	Poor spillover prediction
Livestock interface	Low–moderate	Pigs act as amplifying hosts	Limited routine surveillance	Missed early warning
Environmental drivers	Low	Deforestation and climate variability are implicated	Lack of integrated eco-climate models	Weak risk forecasting
Human transmission	Moderate	Human-to-human transmission documented	Superspreading, asymptomatic spread	Incomplete containment strategies
Clinical spectrum	Low–moderate	Encephalitis-dominant reporting	No standardised severity framework	Limited trial readiness
Diagnostics	Low	PCR-based confirmation	A few field-deployable tools	Delayed outbreak response
Social and behavioural	Low	Sap consumption and caregiving risks	Intervention effectiveness is poorly evaluated	Persistent exposure risks
Governance and ethics	Very low	*Ad hoc* outbreak research	No standardised ethical frameworks	Inequitable access

Among identified gaps, the highest-priority areas include surveillance integration, rapid diagnostics, and understanding viral shedding dynamics, due to their direct relevance to early detection and outbreak containment. In contrast, governance and ethical frameworks, while critical, represent longer-term structural priorities.

**Figure 3:**
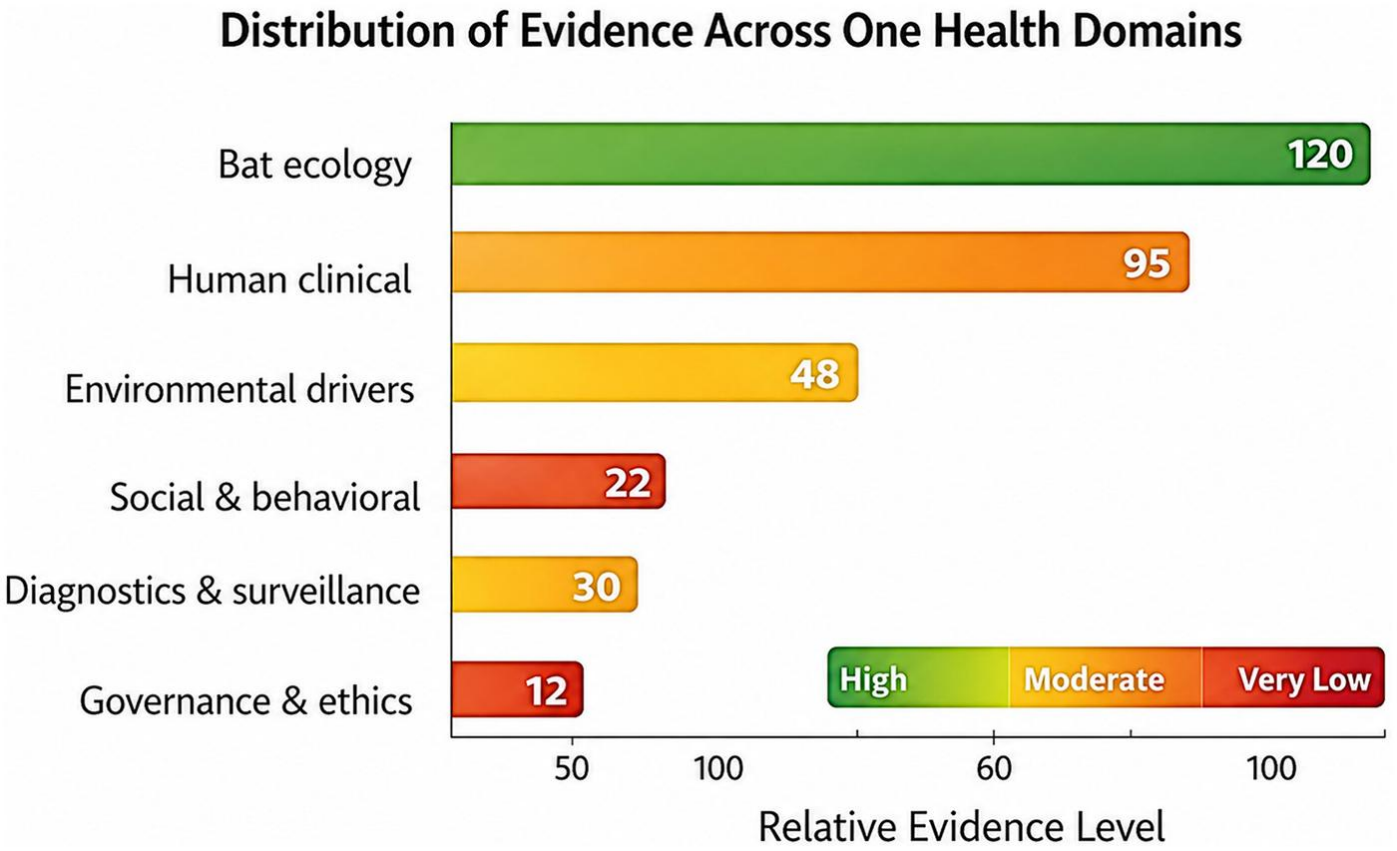
Distribution of evidence across One Health domains. This figure summarises the relative distribution of published evidence across major One Health domains relevant to Nipah virus research. It demonstrates substantial concentration in animal ecology and clinical research, with comparatively limited evidence in governance, social sciences, and surveillance integration.

As illustrated in Figure 3, the distribution of evidence across domains reflects a strong concentration in ecological and clinical research, with comparatively limited studies addressing governance and health systems integration.

## 4 Discussion

A comparative analysis across domains reveals a marked imbalance in the Nipah virus evidence base. Although animal ecology, viral transmission pathways, and clinical manifestations are relatively well characterised, health system preparedness, governance, surveillance integration, and social science research remain underdeveloped. This asymmetry limits the translation of scientific knowledge into operational preparedness and highlights the need for greater investment in implementation science, interdisciplinary collaboration, and One Health operationalisation.

### 4.1 Fragmentation across the human–animal–environment interface

A notable finding of this review is the uneven development of evidence across One Health domains. Research on bat reservoir ecology has advanced considerably, particularly regarding the geographical distribution of *Pteropus* species, viral maintenance, and patterns of host movement (15, 19). More recent longitudinal and modelling studies have improved the understanding of viral shedding dynamics and seasonal pulses associated with ecological stressors (16, 20). Nevertheless, major uncertainties remain regarding the environmental and physiological factors that trigger spillover events, and current evidence remains insufficient to support predictive modelling at a scale suitable for public health preparedness.

Environmental drivers—including deforestation, agricultural intensification, habitat fragmentation, and climate variability—have been consistently implicated in Nipah virus emergence (7, 13, 15–17). However, relatively few studies integrate ecological, climatic, agricultural, and epidemiological variables into unified modelling frameworks. This limitation constrains the development of robust early warning systems capable of anticipating outbreaks before human infections occur. The absence of harmonised datasets linking land-use change, bat ecology, livestock interfaces, and human exposure represents a critical gap in One Health implementation.

### 4.2 Regional and global perspectives on Nipah virus emergence

The epidemiology of Nipah virus exhibits important regional differences that have direct implications for surveillance and preparedness. In Malaysia, the 1998–1999 outbreak was largely driven by transmission from infected pigs, which acted as amplification hosts following spillover from fruit bats (13). In contrast, outbreaks in Bangladesh and India have been characterised predominantly by direct bat-to-human transmission through contaminated date palm sap and subsequent human-to-human transmission within households and healthcare settings (1, 2, 12, 21). These differences highlight the context-specific nature of spillover pathways and the need for tailored intervention strategies.

Globally, Nipah virus shares several ecological and epidemiological characteristics with other bat-borne zoonoses, including Hendra virus and other emerging viral diseases associated with environmental disruption and wildlife–human interfaces. Increasing evidence suggests that land-use change, agricultural intensification, biodiversity loss, and climate variability are important drivers of zoonotic emergence across regions (7, 13, 15, 16, 19). Similar ecological patterns have been observed in bat-associated disease systems worldwide, where habitat alteration influences host behaviour, viral shedding dynamics, and opportunities for pathogen transmission (15–17, 19). These global parallels reinforce the broader relevance of One Health approaches and suggest that lessons learned from Nipah virus preparedness may inform prevention strategies for other emerging zoonotic diseases.

### 4.3 Human health bias and the “Severe Case Lens”

Human Nipah virus research remains heavily focused on severe clinical presentations, particularly acute encephalitis observed during recognised outbreaks (9, 14). This emphasis may underestimate the true burden of infection, as mild, subclinical, or asymptomatic infections are likely underdetected and under-reported. Limited sero-epidemiological evidence further complicates efforts to accurately estimate transmission dynamics, infection fatality rates, and population-level exposure.

The clinical spectrum of Nipah virus infection extends beyond encephalitis to include respiratory disease, systemic manifestations, and delayed neurological relapse (9, 11, 14). However, severity classifications remain poorly standardised across studies and outbreak investigations. The absence of harmonised clinical frameworks limits comparability across outbreaks and complicates the evaluation of therapeutic interventions. Improved clinical characterisation and population-based surveillance are therefore necessary to better define disease burden and support future clinical research.

### 4.4 Surveillance, diagnostics, and governance gaps

Despite repeated outbreaks, Nipah virus surveillance remains largely reactive rather than preventive. Animal, environmental, and public health surveillance systems frequently operate independently, limiting opportunities for early detection and coordinated response. Diagnostic capacity remains highly centralised and dependent on specialised biosafety level-3 or biosafety level-4 laboratory infrastructure, resulting in delayed outbreak confirmation and response, particularly in endemic regions (6, 10). These limitations have been repeatedly identified as barriers to timely containment during outbreak investigations.

Equally under-explored are governance, ethics, and equity considerations. Issues such as data sharing, community engagement, equitable access to diagnostics and future countermeasures, and ethical conduct of outbreak research receive relatively little attention in the current literature despite their importance for public trust and preparedness (18). Strengthening governance frameworks may therefore be as important as advancing laboratory or surveillance capacity.

### 4.5 One Health as concept vs. practice

The One Health framework is frequently invoked in discussions of Nipah virus emergence; however, its implementation often remains conceptual rather than operational. In many settings, One Health serves as a guiding principle without being translated into integrated surveillance systems, interoperable data platforms, or coordinated cross-sectoral response mechanisms. This review demonstrates that effective One Health implementation requires more than interdisciplinary collaboration alone. It necessitates shared indicators, integrated surveillance infrastructures, sustainable financing mechanisms, and the systematic incorporation of social and behavioural sciences into outbreak prevention and preparedness strategies (18).

Without these structural components, One Health risks remaining a conceptual ideal rather than a functional preparedness model. To move from principle to practice, institutions must establish formal mechanisms for collaboration, align incentives across sectors, and integrate ecological and environmental intelligence into real-time public health decision-making. Only through such operationalisation can One Health evolve into a transformative framework for preventing future Nipah virus spillover and other emerging zoonotic threats.

### 4.6 Implementing One Health in low- and middle-income countries

Implementation of One Health approaches remains particularly challenging in low- and middle-income countries (LMICs), where many Nipah virus outbreaks occur. Common barriers include limited laboratory infrastructure, shortages of trained personnel, fragmented governance systems, inadequate surveillance capacity, insufficient funding, and weak mechanisms for intersectoral communication and data sharing (10, 18). These challenges are frequently compounded by competing public health priorities and restricted integration of environmental monitoring into disease surveillance systems.

Several practical strategies could strengthen One Health implementation in resource-constrained settings. Investment in decentralised diagnostic capacity and field-deployable testing platforms would facilitate earlier outbreak detection and response (10). Integrated surveillance systems linking animal, environmental, and human health sectors could improve risk forecasting and early warning capabilities (15, 16, 18, 19). Regional collaboration, cross-border information sharing, and community-based behavioural interventions may further reduce spillover risk and improve preparedness (2, 7). Sustained investment in workforce development, implementation research, and institutional coordination will be essential for transforming One Health from a conceptual framework into an operational preparedness strategy.

### 4.7 Strengths

This review integrates evidence from ecological, environmental, animal, and human health domains to provide a comprehensive One Health perspective on Nipah virus emergence. By synthesising information across disciplines, it highlights critical knowledge gaps that may not be apparent within individual fields of study. The review further emphasises translational challenges, implementation barriers, and preparedness needs, thereby providing a framework for future research priorities and policy development. Inclusion of outbreak investigations, surveillance reports, and grey literature enhances the breadth and practical relevance of the synthesis.

### 4.8 Limitations

Several limitations should be considered when interpreting the findings of this study. First, as a narrative synthesis, the review is inherently susceptible to selection and interpretation bias. Second, the absence of a formal risk-of-bias assessment limits quantitative evaluation of evidence quality. Third, a high level of heterogeneity in study designs, outcomes, and methodologies precludes a formal meta-analysis. Finally, geographic differences in surveillance intensity and data availability may affect the generalisability of findings across regions. Despite these limitations, this review provides a comprehensive cross-sectoral synthesis aligned with One Health principles and identifies key priorities for strengthening preparedness against future Nipah virus outbreaks.

**Figure 4:**
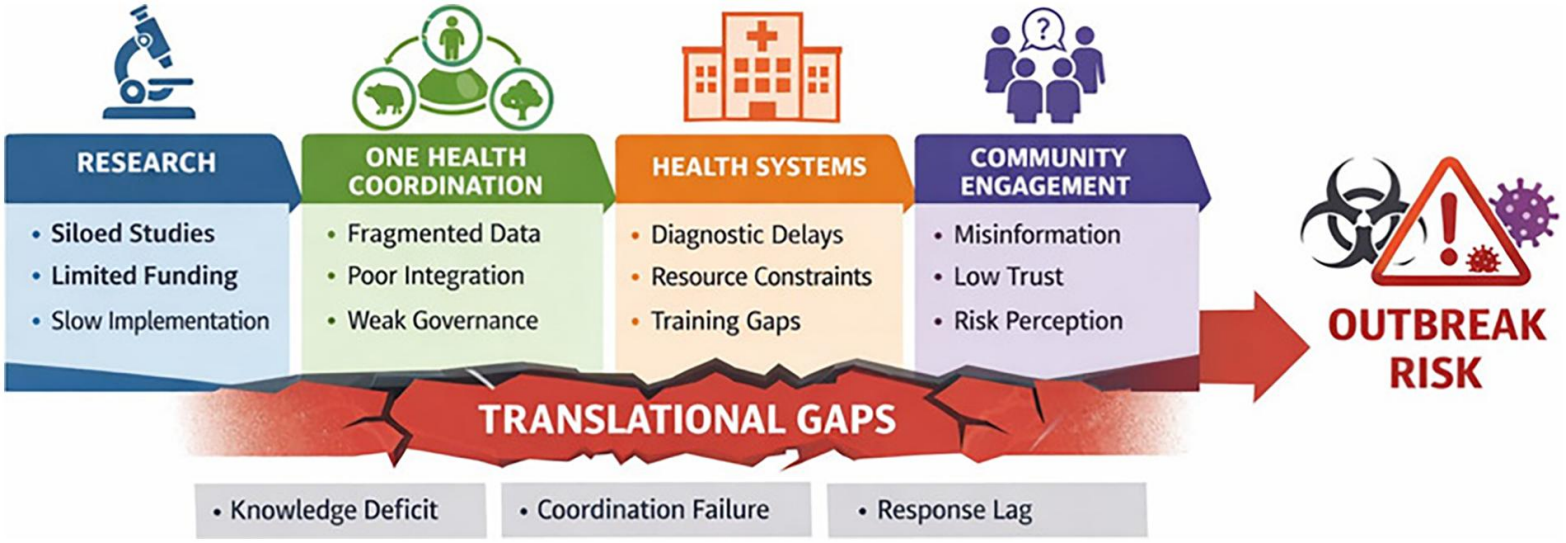
Translational gaps from knowledge to action in Nipah virus preparedness. This figure illustrates the critical bottlenecks that hinder the translation of scientific evidence into effective public health action. Weak data integration, limited predictive modelling, and policy disconnects collectively impede timely and coordinated One Health responses.

The structural bottlenecks limiting translation from research to public health action are illustrated in Figure 4.

### 4.9 Future research agenda: operationalising One Health for Nipah virus

The findings of this review highlight several interconnected research priorities necessary for strengthening Nipah virus prevention and preparedness. Future investigations should prioritise longitudinal studies of bat ecology and viral shedding dynamics to better understand the environmental and physiological drivers of spillover events. Development of integrated eco-epidemiological models that combine climate variability, land-use change, reservoir ecology, and human behavioural factors is essential for improving predictive risk assessment and early warning systems.

Expanded sero-epidemiological studies are needed to quantify asymptomatic infections and refine estimates of disease burden and transmission dynamics. Research should also focus on the development and validation of rapid, field-deployable diagnostic platforms suitable for resource-constrained settings. In parallel, implementation science and behavioural research are required to evaluate community-level interventions, improve risk communication, and enhance adoption of preventive measures. Finally, greater attention should be directed towards governance, ethics, and equitable access to surveillance and response resources, thereby strengthening operational One Health preparedness across human, animal, and environmental health sectors.

Priority areas for operationalising climate-sensitive One Health preparedness are outlined in Table 4.

**Table 4:** Priority research areas for operational One Health implementation.

Priority area	Required approach	Expected outcome
Bat viral ecology	Longitudinal cohort studies	Spillover prediction
Eco-epidemiology	Integrated modelling	Early warning systems
Human serology	Population-based surveys	True burden estimation
Clinical standardisation	Severity classification	Trial readiness
Diagnostics	Point-of-care assays	Rapid containment
Social science	Implementation research	Sustainable prevention
Governance	Ethical frameworks	Trust and equity

## 5 Conclusion

This review demonstrates that substantial progress has been made in understanding Nipah virus ecology, transmission pathways, and clinical manifestations; however, critical knowledge gaps persist across environmental surveillance, spillover prediction, diagnostics, governance, and health system preparedness. The evidence synthesis revealed a pronounced imbalance in research attention, with ecological and clinical investigations substantially outpacing studies focused on implementation, policy translation, and integrated preparedness.

Addressing these gaps will require a transition from fragmented, sector-specific approaches towards fully operational One Health systems that integrate human, animal, and environmental health data into coordinated surveillance and response frameworks. Strengthening ecological forecasting, decentralised diagnostics, integrated surveillance platforms, community engagement, and cross-sector governance mechanisms represents a practical pathway for reducing spillover risk and improving outbreak preparedness.

Future research should prioritise predictive spillover modelling, longitudinal reservoir studies, implementation science, behavioural interventions, and equitable governance frameworks. Advancing these priorities will not only enhance preparedness against Nipah virus but also provide a scalable One Health model for preventing and controlling other emerging zoonotic diseases in an era of accelerating environmental change and increasing pandemic risk.

## Data Availability

The original contributions presented in the study are included in the article/Supplementary Material, further inquiries can be directed to the corresponding author.
